# Editorial: Metallodrugs in cancer therapy

**DOI:** 10.3389/fchem.2025.1758341

**Published:** 2025-12-15

**Authors:** Suman Adhikari, M. Montazerozohori, Anjoy Majhi, Surajit Pathak

**Affiliations:** 1 Department of Chemistry, Govt. Degree College, Dharmanagar, India; 2 Department of Chemistry, Yasouj University, Yasouj, Iran; 3 Department of Chemistry, Presidency University, Kolkata, West Bengal, India; 4 Faculty of Allied Health Sciences, Chettinad Hospital and Research Institute (CHRI), Chettinad Academy of Research and Education (CARE), Chennai, Tamil Nadu, India

**Keywords:** anticancer agents, apoptosis, chemoresistance, metallodrugs, platinum-based drugs

## Introduction

Following cisplatin’s success in the treatment of cancer, numerous other platinum-based complexes have been investigated as potential anticancer drugs, and some of these have received regulatory approval for the purpose of treating cancer. Despite the fact that platinum-based metallodrugs are effective against many different forms of cancer, their widespread usage in treatment is constrained by a number of factors (chemoresistance, poor selectivity, inherent toxicities, etc.) (Romani, 2022). To address the unresolved clinical issues associated with platinum-based metallodrugs, many researchers are focusing on the design and synthesis of metallodrugs with improved biological activity and selectivity, reduced toxicity and alternative mechanisms of action compared to those of platinum-based compounds. Several strategies have been explored to enhance the effectiveness and reduce the adverse effects of metallodrugs. These include the use of various transition metal-based complexes with bioactive ligands that introduce new modes of action, improve delivery, permit selective activation, provide synergistic effects, and enhance tumour accumulation. Metal complexes of ruthenium, gold, copper, iridium, and osmium have been evaluated against various cancer cell lines and are regarded as promising alternatives to Platinum-based drugs, thereby driving substantial interest in the development of new anticancer drugs (Adhikari et al., 2024). The scientific community currently faces a significant challenge in developing potent anticancer therapeutics with high selectivity and minimal toxicity. Owing to their distinctive properties of metals, such as their redox activity, diverse coordination modes, and reactivity towards biomolecules, metal complexes have attracted considerable attention for their therapeutic potential in cancer therapy. These characteristics have made metal complexes desirable candidates for targeting biomolecular targets with specificity, thereby modulating cellularproliferation mechanisms. The goal of this Research Topic is to give readers a broad overview of recently produced metallodrugs used in cancer treatment, with a focus on their past, current, and potential future applications.

This Research Topic, “*Metallodrugs in cancer therapy*” compiles five articles on the latest advances in areas of metal-based complexes for the treatment of cancer.

Mitochondria plays an important role in tumor biology, regulating cellular energy metabolism, free radical generation, apoptosis, autophagy, signal transmission, and other essential biological processes (Nath et al., 2024). The review work of Zheng and co-workers has been focused on the thiosemicarbazone-based metal complexes for cancer therapy through the mitochondrial signalling pathway (Zheng et al.). This review highlights the design and synthesis of various thiosemicarbazone-based metal complexes and their role in anticancer studies. The authors also discussed the structure-activity relationship of thiosemicarbazone scaffolds and highlighted the effects of various substituents attached to thiourea, as well as the role of different metal ions in modulating anticancer activity. The authors attributed that the thiosemicarbazone-based metal complexes have the ability to pass through cell membranes and accumulate in the inner mitochondrial membrane, where they reduce mitochondrial membrane potential and induce apoptosis.

David Morales-Morales and his colleagues investigated theanticancer and antioxidant potential of mononuclear distorted square planar POCOP-Ni (II) pincer complexes synthesized from phloroglucinol (Amaya-Flórez et al.). *In vitro* cytotoxicity studies demonstrated that Ni (II) complexes bearing alkyl groups (^*t*^Bu or ^*i*^Pr) attached to P atoms exhibited strong anticancer activity against various tumor cell lines (U251, K562, HCT-15, MCF-7, and SK-LU-1), with IC_50_ values ranging from ∼2.43 to 7.85 μM. The steric effects of the alkyl moieties on the P atoms may facilitate the release of Chloride (Cl) ions from the coordination sphere, creating a vacant site around the Ni atom that enables interactions with specific biomolecules. Furthermore, molecular docking studies of Ni (II) complexes demonstrated strong interactions with DNA and topoisomerase I.

Celin et al. investigated the photophysical properties and bioactivities of a tris (polypyridyl) Ru (II) complex derived from the hetarocyclic nitrogen donor ligand 4,4′-dimethyl-2,2′-bipyridine (Celin et al.). The anticancer potential of the Ru (II) complexwas assessed through molecular docking studies, which revealed that the interactions were stabilized by π-alkyl contacts and the binding site comprised both hydrophilic and hydrophobic residues. Additionally, the molecular dynamic simulations supported the observed anticancer activity of Ru (II) complex.

Moynihan et al. designed and synthesised four Pt (IV) complexes based on cisplatin, incorporating a glucosamine scaffold in the axial position through conjugated at the C2 site of the sugar ring (Moynihan et al.). These Pt (IV) complexes exhibited strong activity against several cancer cell lines, including glioblastoma (U87), osteosarcoma (U2-OS, SOAS-2, and MG63), and breast cancer (MDAMB 468). Furthermore, Pt (IV) complex drug uptake in 3D MG63 cell lines was greater than cisplatin drug uptake, highlighting the significance of the carbohydrate moiety in drug internalization.

Shan et al. examined parameters impacting survival outcomes and presented a comparative efficacy analysis of oxaliplatin versus irinotecan as first-line therapy in patients with metastatic colorectal cancer who had previously reveived adjuvant treatment (Shan et al.). The authors reported that the survival benefit depends primarily on the effective sequential administration of both oxaliplatin and irinotecan over the course of the disease, rather than on the intrinsic superiority of one agent over the other.

This Research Topic presents a diverse collection of articles on the application of metal complexes in medicine. Researchers in the field of medical bioinorganic chemistry wil find these contributions valuable, as they advance our understanding of the biological properties of metal-based complexes and their potential in anticancer drug development.
